# Illinois State Medical Society

**Published:** 1875-06-01

**Authors:** 


### ILLINOIS STATE MEDICAL SOCIETY.

THE Twenty-Fifth Annual Meeting of the Illinois State Medical Society, was held in the Conservatory of Music, at Jacksonville, Ill., commencing May 18th, a large number of delegates being in attendance. The meeting was called to order by Dr. David Prince, Chairman of the Committee of Arrangements, who welcomed the members to the city of Jacksonville.

Dr. J. H. Hollister, President of the Society, responded in a few appropriate remarks, after which the programme of exercises was announced by Dr. Prince.

On motion of Dr. T. D. Fitch, the Secretary, Dr. W. P. Pierce was chosen Treasurer *pro tem.*, in the absence of Dr. Quine, the regular Treasurer.

On motion of Dr. Prince, the assessment was fixed at three dollars per member.

Dr. Prince also moved, that an initiation fee once paid, should not be demanded again after a period of absence, on the part of any member.

After some discussion, the motion was carried.

Subsequently, on Dr. Pierce asking for information, as to whether if a member had paid an initiation fee twenty years ago, and had been absent the intervening years, he was still to be considered a member, Dr. Fitch moved a reconsideration of the motion of Dr. Prince, which was carried—ayes 16; noes 10.

On motion of Dr. Fitch, the order was so amended, that a member loses his membership if he does not attend the meetings of the Society, or pay his dues for a period of five years.

The Standing Committees were next called in their order, and the time fixed to hear such reports as were ready.

Dr. Whitmire, Chairman of the Committee on Nervous Diseases, announced that he had requested Dr. J. S. Jewell to prepare the report, and that he had prepared one.

The Secretary announced that Dr. Charles G. Smith, of Chicago, Chairman of the Committee on Dermatology, had not prepared a report, but desired to be continued on the committee another year. The request was granted.

A report of Dr. J. N. Niglas, of Peoria, on Hernia, was referred to the Committee on Surgery.

Dr. J. W. Freer announced a volunteer paper on the subject of Transfusion of Blood.

Dr. Hoyt, of Bloomington, announced a paper prepared by Dr. E. W. Grey, on Small-Pox, together with a series of resolutions on Vaccination, which he asked the Society to adopt. It was referred to the Committee of Arrangements.

Dr. Rumbold, of St. Louis, announced a volunteer paper on the Functions of the Uvula, which was ordered to be read at the convenience of the Society.

The Secretary announced that Prof. L. Curtis, of Chicago, had prepared a paper on the Anatomy of the Serous Membranes, as affecting their Pathology. It was ordered to be read.

A communication was read from the Æsculapian Medical Society, requesting that Dr. William Massey's Essay on Insanity, be read before the Illinois State Medical Society. That gentleman was accordingly invited to read his paper, and this was the first paper read.

It was followed by the reading of Dr. J. B. Walker's Essay on Scarlatina, which was referred to the Committee on Publication.

The next thing was the reading of the paper of Dr. Curtis', on the Anatomy of the Serous Membrane.

After some discussion of the subject by Drs. J. W. Freer, Curtis, and Worrell, the paper was referred to the Committee on Publication.

On motion of Dr. Worrell, the paper of Dr. Massey was referred to the Committee on Publication.

In the afternoon, at 2 o'clock, the Association visited the Deaf and Dumb Asylum, and the Institution for Feeble-Minded Children, spending the time there until 5 o'clock. At 7½ o'clock in the evening, the Society re-assembled, and Dr. J. O. Hamilton, of Jerseyville, read a paper on the Diseases of Children, which was followed by one on the same subject, prepared by Dr. C. W. Earle, of Chicago, read by the Secretary.

After a pretty full discussion of the papers by Drs. E. F. Ingalls, Hamilton, Steele, Fitch, J. H. Mosier, Slater, Gowen, Worrell, Rumbold, and R. C. Hamill, the papers were referred to the Committee on Publication.

Dr. Rumbold, of St. Louis, read his paper on the Functions of the Uvula, which was referred to the Committee on Publication.

Dr. David Prince next delivered an Address on the Use of Electricity in Therapeutics and Surgery, accompanying the address by experiments with electrical machines. He was requested to prepare a paper on the subject for publication in the transactions.

On motion of Dr. Freer, the proposition of Prof. Sanders of the Conservatory of Music, to entertain the Society with a concert, was accepted, and the time fixed for Wednesday evening.

Adjourned.

---

WEDNESDAY, May 19.

The Society was called to order at 8 o'clock.

Dr. J. W. Freer read his paper on Transfusion, which was referred to the Committee on Publication.

Dr. T. D. Fitch announced that, in connection with the Report of the Committee on Obstetrics, he would present some remarks on several new gynæcological instruments.

A recess of ten minutes was then taken, to allow of the selection of a Nominating Committee. The Committee chosen were as follows:

J. W. Freer, M.D., Cook county; J. H. Mosier, M.D., Whiteside county; D. E. Foote, M.D., Boone county; T. N. Stewart, M.D., Scott county; Thomas H. Whitten, M.D., Montgomery county; M.W. Seaman, M.D., Macoupin county; M. W. Walton, M. D., Stephenson county; F. B. Haller, M. D., Fayette county; E. Wenger, M.D., Iroquois county; D. Lichty, M. D., Ogle county; L. B. Slater, M.D., Christian county; W. A. Haskill, M.D., Madison county; A. P. Tenney, M.D., McLean county; Fred. Cole, M.D., Woodford county; D. L. Wood, M.D., La Salle county; Samuel Mileham, M. D., Adams county; P. H. Garretson, M.D., McDonough county; T. J. Maxton, M. D., Henderson county; G. W. Albin, M.D., Cumberland county; W. L. Goodell, M. D., Effingham county; A. F. Darrah, M.D., Champaign county; J. S. Williams, M.D., Jersey county; Geo.W.Wright, M.D., Fulton county; W. H.Veatch, M.D., Green county; J. G. McKinney, M.D., Pike county; W. G. Cochran, M.D., De Witt county; J. V. Frazier, M.D., Mercer county; C. T. Wilbur, M.D., Morgan county; J. N. Allen, M.D., Brown county; J. L. Hayes, M.D., Edgar county; D. H. Alris, M.D., Henry county.

After the recess, Dr. E. B. Cooke submitted his report on Practical Medicine, which was followed by a paper by Dr. R. C. Hamill on the Treatment of Rheumatism by phytolacca and guiaca. The papers were discussed by Drs. Steele, Cooke, Birney, Herriott, Wright, Hay, and Edgar of St. Louis, and were referred to the Committee on Publication.

Dr. E. Powell next read the report of the Committee on Surgery, which was followed by one on Hernia, by Dr. E. L. Herriott.

Dr. David Prince exhibited an instrument for taking up the stitch in operations on soft palate and in skin planting. Adjourned.

In the afternoon the Society met, at 2 o'clock, to witness the operation of transfusion performed upon a dog by Dr. J. W. Freer, aided by Drs. E. Powell and Walter Hay.

At the conclusion of this the members took the street cars, and visited the State Hospital for the Insane, and the Asylum for the Blind.

In the evening the President, Dr. J. H. Hollister, delivered his annual address before a large audience, the platform being occupied by the Ex-Presidents present, Drs. Hamilton, McFarland, Worrell, Steele, Haller, and Prince. At the conclusion of the address, Dr. Prince offered a resolution expressing the thanks of the citizens of Jacksonville for the pleasure they had in listening to the eloquent and scholarly address of the President. The resolution was unanimously adopted. The remainder of the evening was devoted to a concert given by the Illinois Conservatory of Music.

THURSDAY, May 20.

The Association re-assembled at 8 A. M.

The paper of Dr. Niglas, on Hernia, was referred to the Committee on Publication.

Dr. W. P. Pierce read a paper on Fractures, which was referred to the committee.

Dr. Haller, chairman of the Nominating Committee, submitted his report, which, after some discussion and amendment by the committee, was submitted as follows:

REPORT OF THE NOMINATING COMMITTEE—OFFICERS FOR THE ENSUING YEAR.

President, Thomas D. Washburn, M.D., Hillsboro; First Vice-President, J. L. White, M.D., Bloomington; Second Vice-President, John Wright, M.D., Clinton; Treasurer. John H. Hollister, M.D., Chicago; Per. Secretary, T. Davis Fitch, M.D., Chicago; Ass't Secretary, C. B. Johnson, M.D., Tolona.

STANDING COMMITTEE.

*Board of Censors.*—E. Wenger, M.D., Gilman, *Chairman*; E. Ingals, M.D., Chicago; E. L. Herriott, M.D., Grafton.

*Committee of Arrangements.*—S. H. Birney, M.D., Urbana, *Chairman*; A. F. Darrah, M.D.; J. T. Pearman, M.D., Champaign.

*Practical Medicine.*—Wm. Massey, M.D., Grand View, *Chairman*; D. L. Jewett, M.D., Watseka; J. W. Fink, M.D., Hillsboro.

*Committee on Surgery.*—Moses Gunn, M.D., Chicago, *Chairman*; A. C. Rankin, M.D., Loda; John G. Hays, M.D., Paris.

*Committee on Obstetrics.*—M. W. Walton, M.D., Ridott, *Chairman*; C. Goodbrake, M.D., Clinton; D. E. Foote, M.D., Belvidere.

*Drugs and Medicines.*—J. H. Etheridge, M.D., Chicago, *Chairman*; T. J. Pitner, M.D., Jacksonville; H. H. Hood, M.D., Litchfield.

*Necrology.*—G. W. Albin, M.D., Neoga; Wm. Chambers, M. D., Charleston; J. N. Niglas, M.D., Peoria.

*Ophthalmology.*—E. L. Holmes, M.D., Chicago.

*Otology.*—F. C. Hotz, M.D., Chicago.

SPECIAL COMMITTEES.

*Diseases of Children.*—J. L. Williams, Otterville.

*Medical Jurisprudence.*—M. A. McClellan, M.D., Knoxville.

*Dermatology.*—J. N. Hyde, M.D., Chicago.

*Progress in Physiology.*—Sarah Hackett Stevenson, M.D., Chicago.

*Idiocy.*—C. T. Wilbur, Jacksonville.

*Neurology.*—Walter Hay, M.D., Chicago.

*Gynæcology.*—A. Reeves Jackson, M.D., Chicago.

*Hygiene.*—John Little, M.D., Leroy.

*Diagnosis and Extirpation of Tumors.*—W. L. Goodell, M.D., Effingham.

*Empiricism.*—J. F. Todd, M.D., Galva.

*Prudence in the Administration of Therapeutic Agents.*—E. Ingals, M.D., Chicago.

*Uterine Displacements.*—F. D. Fitch, M.D., Chicago.

*Staphyloraphy*—David Prince, M.D., Jacksonville.

*Electro-Therapeutics.*—Plym S. Hayes, M.D., Chicago.

*Place of Meeting.*—Urbana, Champaign Co.

The report was adopted.

Dr. T. D. Fitch submitted some new gynæcological instruments, with explanations. A discussion followed, which was participated in by Drs. Cooke, Edgar, of St. Louis, Frazier, and Boal.

Dr. David Prince also exhibited several similar instruments with explanations.

Dr. Pierce read an abstract of a report on Surgery. The report was referred to the Publishing Committee.

Dr. E. Ingals, Chairman of the Committee, submitted his report on Necrology. It included memoirs of Drs. G. W. Young, of Aurora, and J. V. Z. Blaney, of Chicago.

The report on Nervous Diseases, by Dr. J. S. Jewell, was next received and referred to the Publication Committee.

Dr. Prince declining to serve on the Committee on Galvano-Therapeutics for next year, his resignation was accepted. Dr. William Hay, of Chicago, was appointed to prepare a paper on Electro-Therapeutics.

Resolutions were adopted requesting Congress to place the medical corps of the army of the United States on an equal footing with the officers of other army corps.

Dr. T. D. Fitch read a report prepared by Dr. H. A. Johnson, of Chicago, on Phthisis, which was referred to the Committee on Publication.

The Secretary exhibited an improved stethoscope, which Dr. Curtis explained.

The resolutions proposed by Dr. E. W. Gray, of Bloomington, on the regulation, by a State enactment, of the subject of vaccination in all public schools, asylums, etc., were read and adopted.

On motion of Dr. Haller, Dr. Prince was appointed a special committee on Staphyloraphy.

The Secretary submitted a report of the members present, and the Society adjourned until 2½ o'clock in the afternoon.

The Treasurer's Report was read and accepted.

Also the Secretary's Report, and the report of the Committee on Publication.

On motion of Dr. Freer, the Secretary was voted a salary of $100.

After some discussion in regard to the delay in printing the transactions, Dr. Hay submitted the following, which was adopted:

*Resolved*, That at the next and at all subsequent meetings, all reports of committees, and other papers designed for publication in the transactions of this Society, be furnished in form ready for publication, and placed at the disposal of the Publication Committee.

The following named persons were appointed delegates to the next session of the American Medical Association:

S. C. Plummer, M.D., Rock Island; Walter Hay, M.D., Chicago; J. F. Todd, M.D., Galva; J. B. Rood, M.D., Lemont; D. E. Foote, M.D., Belvidere; F. B. Haller, M.D., Vandalia; J. W. Freer, M.D., Chicago; J. H. Hollister, M.D., Chicago; W. P. Pierce, M.D., Lemont; Moses Gunn, M.D., Chicago; N. S. Reed, M.D., Chandlerville; H. B. Buck, M.D., Springfield; Thomas J. Whitten, M.D., Irving; Wm. L. Goodell, M.D., Effingham; David Prince, M.D., Jacksonville; G. Wheeler Jones, M.D., Danville; J. Adams Allen, M.D., Chicago; E. P. Cook, M.D., Mendota; A. F. Darrah, M.D., Urbana; M. W. Walton, M.D., Ridott; M. Sheppard, M.D., Pason, Adams Co.; C. Goodbrake, M.D., Clinton; J. O. Hamilton, M.D., Jerseyville; Lester Curtis, M.D., Chicago; T. D. Fitch, M.D., Chicago; E. R. Willard, M.D., Wilmington; Wm. A. Haskill,

M.D., Edwardsville; Robert Boal, M.D., Peoria; C. T. Wilbur, M.D., Jacksonville; J. M. Steele, M.D., Grand View; L. Clark, M.D., Rockford; O. Everett, M.D., Dixon; T. D. Washburn, M.D., Hillsboro; Wm. A. Elder, M.D., Bloomington; C. Paul Simon, M.D., Chicago; E. Ingals, M.D., Chicago.

*Iowa.*—Walter Hay, M.D., Chicago; T. D. Fitch, M.D., Chicago.

*Missouri.*—F. B. Haller, M.D., Vandalia.

*Indiana.*—W. W. McMann, M.D., Gardner.

*Minnesota State Medical Society.*—L. C. Plumm, M.D., Rock Island.

*New York State Medical Society.*—W. P. Pierce, M.D., Lemont.

Resolutions expressive of the gratification of the members with their treatment by the citizens of Jacksonville were adopted, also a resolution thanking the President, Dr. Hollister, for the skill and wisdom he had displayed as presiding officer, was unanimously adopted, and, after a few farewell words from the President, the Society adjourned.

